# Transcatheter Aortic Valve Implantation Outcomes and Challenges in Asia: A Systematic Review and Meta-Analysis

**DOI:** 10.31083/j.rcm2403079

**Published:** 2023-03-06

**Authors:** Frederick Berro Rivera, Deogracias Villa De Luna, Marie Francesca Mapua Ansay, Ryan T. Nguyen, Gabrielle Pagdilao Flores, John Vincent Magalong, Sung Whoy Cha, John Paul Aparece, Jacques Simon T. Gonzales, Wailea Faye C. Salva, Gerard Francis E. Mangubat, Mer Lorraine P. Mahilum, Taku Inohara, Krishnaswami Vijayaraghavan, Fareed Moses S. Collado, Azeem Latib

**Affiliations:** ^1^Department of Medicine, Lincoln Medical Center, New York, NY 10451, USA; ^2^Department of Internal Medicine, Danbury Hospital, Danbury, CT 06810, USA; ^3^Ateneo de Manila School of Medicine and Public Health, 1604 Pasig, Philippines; ^4^Department of Medicine, Houston Methodist, Houston, TX 77030, USA; ^5^King George Hospital, IG3 8YB Ilford, UK; ^6^Department of Medicine, San Beda University College of Medicine, 1005 Manila, Philippines; ^7^Cebu Institute of Medicine, 6000 Cebu, Philippines; ^8^Southern Philippines Medical Center, 8000 Davao, Philippines; ^9^Department of Cardiology, Keio University School of Medicine, 160-8582 Tokyo, Japan; ^10^University of Arizona, Tucson, AZ 85721, USA; ^11^Department of Cardiology, Rush University Medical Center, Chicago, IL 60612, USA; ^12^Section of Interventional Cardiology-Structural Heart, Montefiore Medical Center, Albert Einstein College of Medicine, New York, NY 10461, USA

**Keywords:** transcatheter, aortic valve, aortic stenosis, TAVR, TAVI, outcomes, Asia

## Abstract

**Background:**

Aortic stenosis (AS) is the world’s most prevalent heart valve disease. Transcatheter aortic valve replacement (TAVR) or Implantation (TAVI) is widely available yet adopting this procedure in Asia has been slow due to high device cost, the need for specific training programs, and the lack of specialized heart teams and dedicated infrastructures. The limited number of randomized controlled trials describing TAVI outcomes among the Asian population hampered the approval for medical reimbursements as well as acceptance among surgeons and operators in some Asian countries.

**Methods:**

A comprehensive medical literature search on TAVI and/or TAVR performed in Asian countries published between January 2015 and June 2022 was done through MEDLINE and manual searches of bibliographies. The full text of eligible articles was obtained and evaluated for final analysis. The event rates for key efficacy and safety outcomes were calculated using the data from the registries and randomized controlled trials.

**Results:**

A total of 15,297 patients were included from 20 eligible studies. The mean patient age was 82.88 ± 9.94 years, with over half being females (62.01%). All but one study reported Society of Thoracic Surgeons (STS) scores averaging an intermediate risk score of 6.28 ± 1.06%. The mean logistic European Systems for Cardiac Operations Risk Evaluation (EuroSCORE) was 14.85. The mean baseline transaortic gradient and mean aortic valve area were 50.93 ± 3.70 mmHg and 0.64 ± 0.07 ${\mathrm{cm}}^{2}$, respectively. The mean procedural success rate was 95.28 ± 1.51%. The weighted mean 30-day and 1-year all-cause mortality rate was 1.66 ± 1.21% and 8.79 ± 2.3%, respectively. The mean average for stroke was 1.98 ± 1.49%. The acute kidney injury (AKI) rate was 6.88 ± 5.71%. The overall major vascular complication rate was 2.58 ± 2.54%; the overall major bleeding rate was 3.88 ± 3.74%. Paravalvular aortic regurgitation rate was 15.07 ± 9.58%. The overall rate of pacemaker insertion was 7.76 ± 4.6%.

**Conclusions:**

Compared to Americans and Europeans, Asian patients who underwent TAVI had lower all-cause mortality, bleeding, and vascular complications, however, had a higher rate of postprocedural aortic regurgitation. More studies with greater sample sizes are needed among Asian patients for a more robust comparison.

## 1. Introduction

Aortic stenosis (AS) is the most prevalent heart valve disease worldwide [1, 2]. In Western countries, transcatheter aortic valve replacement (TAVR) or implantation (TAVI) has become a widely available and standardized procedure, such that the number of patients undergoing TAVI has surpassed the number of patients undergoing surgical AV replacement (SAVR) for AS each year over the last few years [3]. Since the birth of TAVI, the advancement of technology has paved the way for its rapid expansion and will most likely attain an “all-risk” indication [4, 5, 6]. TAVI procedures were done in Asia two years after it was introduced in Europe and the United States [7]. The first TAVI procedure was done in Singapore and since then it has been embraced across the rest of the Asian region [7, 8]. TAVI was also introduced later in China, with its use increasing rapidly due to the rising evidence of efficacy and safety from observational studies and randomized trials [9]. Although TAVI is expanding in western countries, implementation of this modality in some regions in Asia has been slow [7]. This is mainly driven by factors such as cost, paucity of centers that offer advanced training, and inaction from the government sectors [7]. Furthermore, it is difficult to lobby for procedural reimbursements resulting in patients using their own money to pay for the procedure.

This meta-analysis aims to evaluate the efficacy and safety outcomes of TAVI in Asia. For this purpose, we provide information about the key findings generated from Asian TAVI registries and clinical trials. Finally, we compare TAVI outcomes in Asia to the recent data from the US and other Western countries.

## 2. Materials and Methods

### 2.1 Data Sources and Study Selection

**Fig. 1.**
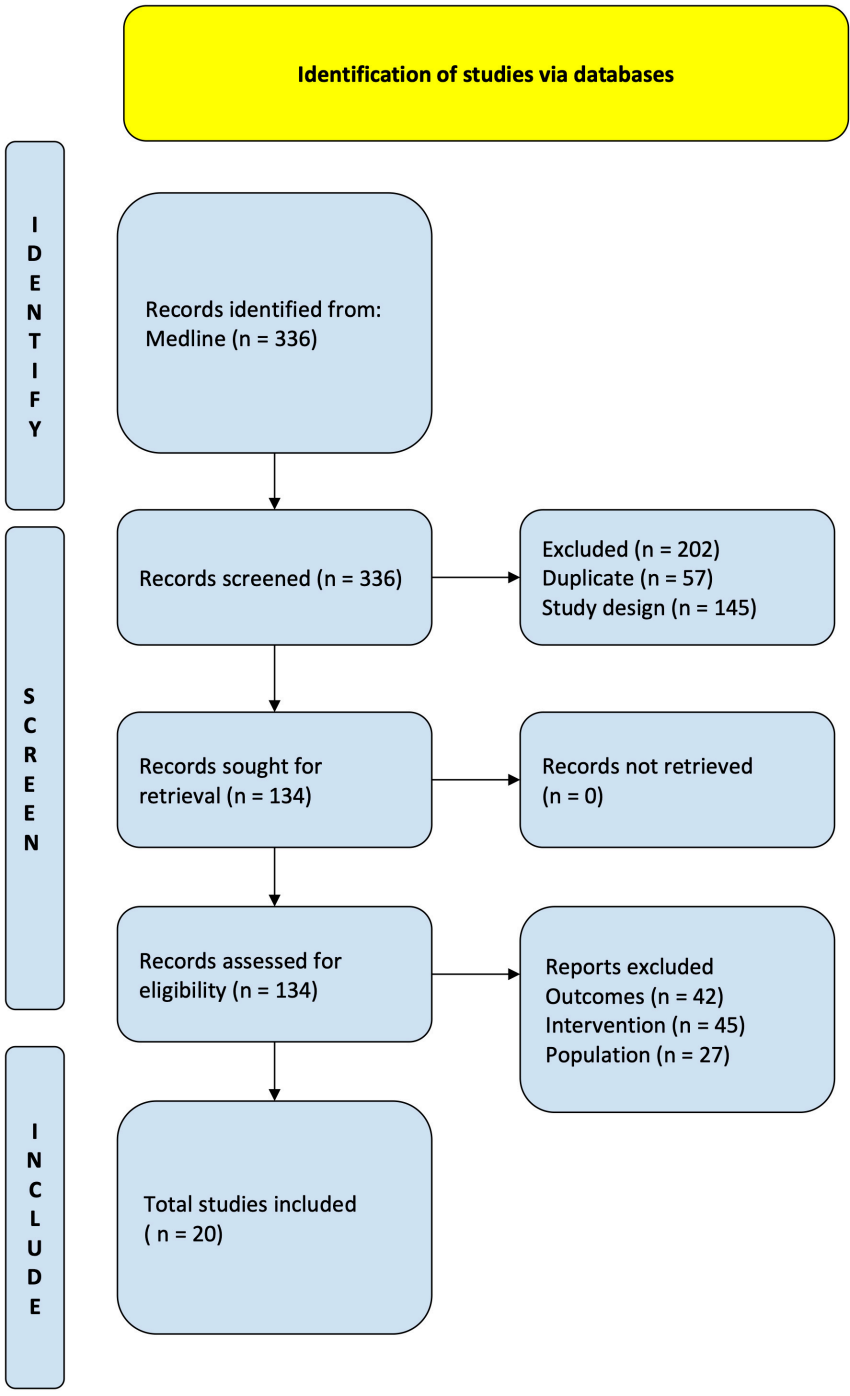
**PRISMA flow diagram**. Study flow based on the Preferred Reporting Items for Systematic review and Meta-Analysis Protocols (PRISMA-P).

This study was first registered in the International Prospective Register of Systematic Reviews (PROSPERO), with the ID number CRD42022359895 [10]. Two independent investigators did a comprehensive search of the medical literature using the MEDLINE database to identify all studies on TAVI was conducted. Articles from inception to July 2022 were included. Search terms include but are not limited to “TAVI”, “TAVR”, “transcatheter”, “transfemoral”, “percutaneous”, “aortic valve”, “replacement”, “Implantation”, “Asia”, “Japan”, “Korea”, “China”, “Vietnam”, “Thailand”, “India”, and other Asian countries. Relevant keywords and their combinations were applied in the search strategy and limited to results in the English language. Manual searches of the bibliography of relevant papers supplemented the search strategy. The multistage was used to determine inclusion for analysis. The eligibility criteria for inclusion of studies are the following: randomized controlled trials or observational cohort studies (both retrospective and prospective) of adults aged >18 years who underwent TAVI in Asian centers, and reports that provide a description of the pacemaker status. Abstracts were reviewed, and studies done on the same registry were considered duplicates. Studies designed as case reports, systematic reviews, and meta-analyses were excluded. The full texts of subsequent articles were obtained and reviewed for data extraction. Studies were evaluated and weighed on the total number of patients included in the analysis. Data to be collected include last name of first author, year of publication, study type, study period, country where TAVR was performed, total population, mean age, percentage of males and females, cardiac and non-cardiac risk factors, baseline scores for risk of cardiac mortality, baseline echocardiographic results, and post-TAVI outcomes. Those with missing data were excluded from analysis. This protocol was designed based on the Preferred Reporting Items for Systematic Review and Meta-Analysis Protocols (PRISMA-P) Statement, presented in Fig. 1 [11, 12]. The Newcastle-Ottawa Scale (NOS) tool was used to assess the risk of bias in non-randomized studies [13, 14]. Components of this scale include the selection of cases and controls, comparability of cases and controls, and outcome follow-up. Two independent reviewers made judgments, and disagreements were resolved through discussion.

### 2.2 Selection of Study Outcomes

This study adopted the Valve Academic Research Consortium 2 (VARC-2) Scale to evaluate post-TAVI outcomes [15]. It includes perioperative complications including, but not limited to, stroke, acute kidney injury, and bleeding. In this paper, TAVI will refer to both TAVR and TAVI. Stroke is the sudden onset, localized or widespread neurological impairment due to damage from vascular infarct or hemorrhage. Closely related is transient ischemic attack (TIA), defined as any reversible neurological deficit lasting less than 24 hours. For this review, acute kidney injury (AKI) is characterized by changes in serum creatinine and urine output and following the diagnostic criteria of AKI in the VARC-2, it was extended from 72 hours to 7 days as a follow-up renal function assessment is done after seven days for patients until stabilization of the condition. Despite being rare, periprocedural myocardial infarction (MI) is also assessed. Bleeding is defined by the VARC-2 using the Bleeding Academic Research Consortium (BARC) criteria and staging. Procedural success, or device success, is defined by VARC-2 as the correct positioning of the prosthetic valve in its proper location, performing as it is intended, and without procedural mortality. Paravalvular leaks (PVL) is one of the most common complications of TAVI and have been associated with poor short-term and long-term outcomes. Atrioventricular blocks (AVB), which may require permanent pacemaker implantation, may occur from mechanical trauma or inflammation caused by the TAVI valve on the conduction system.

### 2.3 Statistical Analysis

Event rates were calculated as the total number of events/occurrences in the studies divided by the total number of patients in the studies with available data. The approach to calculating individual rates for different studies and combining them yields identical results if the weights are defined as the proportion of patients in a study. Results were tabulated as weighted mean ± standard deviation with 95% confidence intervals (CI). Forest plots were generated to compare an outcome post-TAVI across each study. The $I^{2}$ test was used to assess statistical heterogeneity, wherein $I^{2}$ greater than 50% indicated a high degree of heterogeneity. The software Medcalc version 20.118 (MedCalc Software Ltd, Ostend, Belgium) was used for all analyses.

## 3. Results

### 3.1 Study Selection

**Fig. 2.**
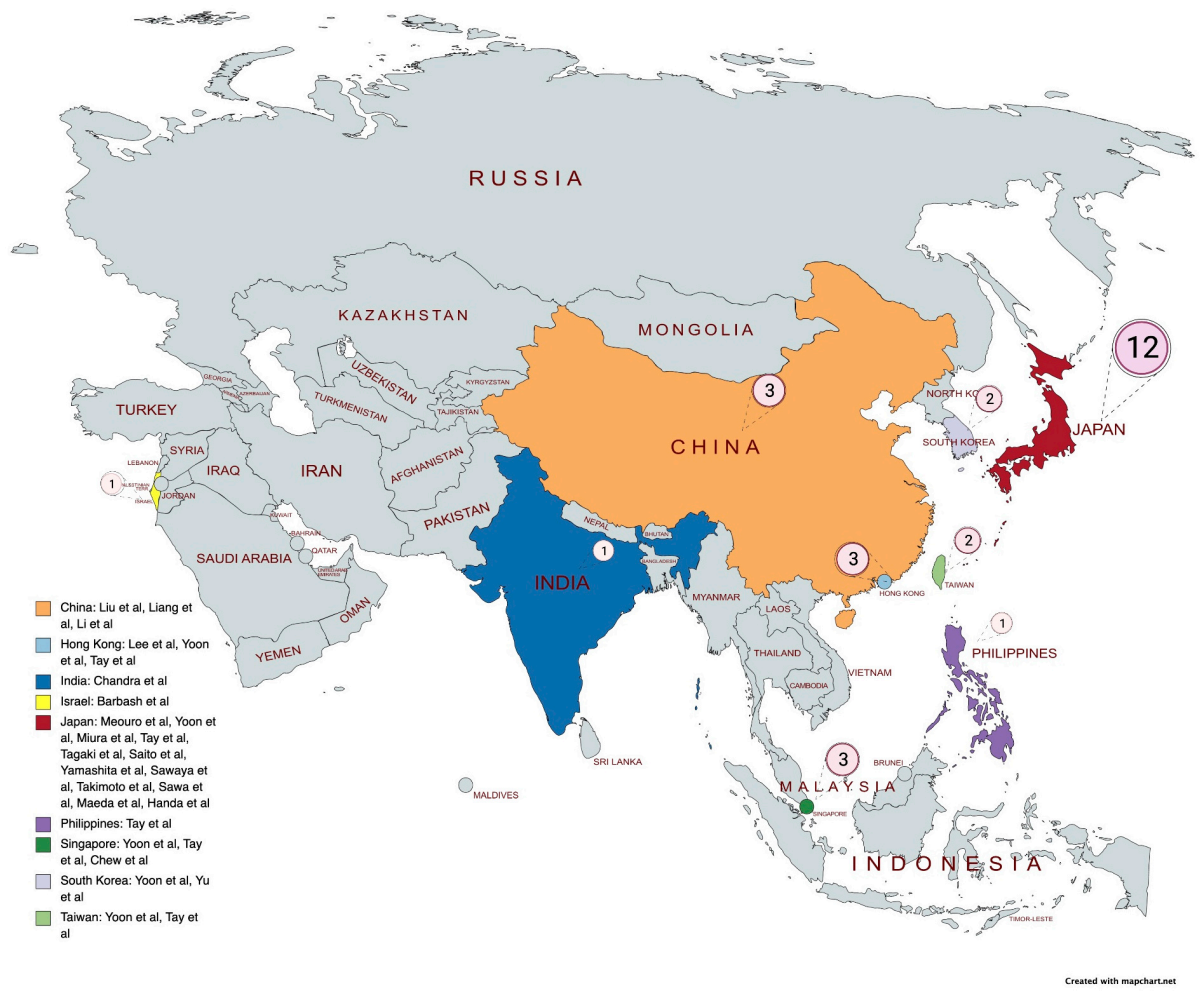
**Distribution of studies**.

A comprehensive literature search identified 336 citations published within the predetermined time span of the search from January 2010–August 2022. After careful review, twenty studies comprising 15,295 patients undergoing TAVI met the study criteria and were selected for the current analysis. The registries and studies included data from Hong Kong, Japan, the Philippines, Singapore, Taiwan, South Korea, India, Israel, and China (see Fig. 2). An overview of the studies is provided in Table 1 (Ref. [8, 16, 17, 18, 19, 20, 21, 22, 23, 24, 25, 26, 27, 28, 29, 30, 31, 32, 33, 34]). Most of these studies had a moderate risk of bias in accordance with the Newcastle-Ottawa Scale, as shown in **Supplementary Table 1**.

**Table 1.** **Summary of studies**.

Author (Year)	Study type	Study period	Country	N	Mean age	Male (%)	Female (%)	Logistic EuroSCORE	Mean STS score (%)
Lee (2017) [16]	Prospective	2010–2015	Hongkong	56	81.9 ± 4.8	64.3	36.7	22.6% ± 13.4%	7.0 ± 4.4
Meguro (2021) [17]	Prospective	2013–2017	Japan	5870	85 (82–88)	6.8	93.2	12.8 (9.3–21.6)	6.7 (4.9–9.3)
Yoon (2016) [18]	Prospective	2010–2016	Singapore, Hong Kong, Taiwan, Japan, Korea	848	81.8 ± 6.6	46.7	53.3	16.5 ± 12.0	5.2 ± 3.8
Miura (2017) [32]	Prospective	2013–2015	Japan	112	84.5 ± 6.6	33.9	66.1	16.0 (11.0–23.0)	6.0 (4.0–9.0)
Liu (2018) [19]	Prospective	2014–2015	China	43	73.9 ± 5.7	69.8	30.2	25.5% ± 5.3%	-
Tay (2021) [8]	Prospective	2009–2017	Hong Kong, Japan, Philippines, Singapore, Taiwan	1125	79.9 ± 8.1	48.5	51.5	20.4 ± 16.7	7.1 ± 6.2
Chandra (2021) [33]	Prospective	2016–2018	India	40	74.5 ± 6.7	60	40	-	5.6 ± 4.2
Takagi (2020) [34]	Prospective	2013–2016	Japan	1613	84.4 ± 5.1	29.6	70.4	17.0 ± 13.1	8.3 ± 7.0
Saito (2021) [20]	Prospective	2015–2016	Japan	50	84.0 ± 6.0	40	60	4.6 ± 4.3	6.4 ± 2.9
Yu (2018) [21]	Prospective	2015–2017	Korea	576	79 (75–83)	48.6	51.4	5.0 (2.0–15.0)	5.2 (3.0–9.0)
Yamashita (2019) [22]	Prospective	2016–2017	Japan	11	83 (80–86)	27.3	72.7		7.2 (5.4–9.8)
Sawa(2017) [23]	Prospective	2010–2011	Japan	64	84.3 ± 6.1	34.4	65.6	-	9.0 ± 4.5
Takimoto (2016) [25]	Prospective	2013–2015	Japan	302	85.0 ± 5.6	34.1	65.9	-	7.4 ± 5.3
Chew (2017) [26]	Prospective	2010–2015	Singapore	59	76.8 ± 8.7	61	39	18.7 ± 15.3	6.9 ± 5.8
Liang (2021) [27]	Prospective	2012–2018	China	175	76.6 ± 5.84	59.4	40.6	-	2.67 (1.76, 3.8)
Sawa (2014) [24]	RCT	2011–2012	Japan	55	82.5 ± 5.5	30.9	69.1	21.5 ± 9.9	8.0 ± 4.2
Maeda (2015) [28]	Prospective	2013–2014	Japan	15	83.3 ± 6.0	26.7	73.3	21.9% ± 11.6%	7.5 ± 3.1
Barbash (2015) [29]	Prospective	2008–2014	Israel	1327	83 (79–86)	43	57	14.24 (9.2-23.6)	4.4 (3.1–6.6)
Li (2021) [30]	Prospective	2012–2020	China	1202	73.8 ± 6.5	57.2	42.8	-	6.0 (3.7–8.9)
Handa (2018) [31]	Prospective	2013–2015	Japan	1752	85 (81–88)	30.5	69.5	-	6.5 (4.5–9.3)

Abbreviations: EuroSCORE, European Systems for Cardiac Operations Risk Evaluation; STS Score, Society of Thoracic Surgeons Score; N, population.

### 3.2 Study Population

Characteristics of the patient population are summarized in Table 2a. The mean patient age was 82.88 ± 9.94 years, with over half being females (62.01%). Two risk stratification models for cardiac surgery patients were used—the Society of Thoracic Surgeons (STS) model, a widely accepted scoring system that is said to be a complete system and takes into account several outcomes such as stroke, renal failure, and length of hospital stay; and the Logistics European System for Cardiac Operative Risk Evaluation (EuroSCORE), which is easier to use due to fewer variables incorporated but is more likely to underestimate or overestimate risks for cardiac surgery patients. All but one study reported Society of Thoracic Surgeons (STS) scores averaging an intermediate risk score of 6.28 ± 1.06%. The mean logistic EuroSCORE reported was 14.85. All but one study reported heart failure New York Heart Association (NYHA) functional class III/IV in their patients, and less than half (35.87 ± 10.52%) of the included patients had heart failure. Prevalence of cardiovascular comorbidities, such as coronary artery disease (CAD), peripheral arterial disease (PAD), history of coronary artery bypass grafting (CABG), and history of percutaneous coronary intervention (PCI) are also summarized in Table 2a. Cerebrovascular disease was reported in all but one study, and prevalence averaged 10.74 ± 6.85%. All but one study reported chronic obstructive pulmonary disease (COPD) and diabetes mellitus. Most of the studies reported hypertension and chronic kidney disease. The mean prevalence of hypertension was high at 75.9% (11,523 out of 15,181 patients). Dyslipidemia was reported in only three of the included publications, involving 6368 of 12,269 patients, and 51.90 ± 11.36% had dyslipidemia.

**Table 2a.** **Patient characteristics**.

Characteristics	No. of publications with data	Overall no. of patients	No. of events	Weighted mean
Age (years)	20	15,295	N/A	82.88 ± 9.94
Male gender (%)	20	15,295	5666	37.04 ± 9.94
Female gender (%)	20	15,295	9484	62.01 ± 1.06
STS score, %	19	15,252	N/A	6.28 ± 1.06
Logistic EuroSCORE, %	12	11,187	N/A	14.85 ± 2.71
Logistic EuroSCORE II, %	4	3991	N/A	4.80 ± 0.65
NYHA 1 and 2 (%)	19	13,968	8417	60.26 ± 16.00
NYHA 3 and 4 (%)	19	13,968	5548	39.72 ± 16.01
CAD (%)	19	15,120	5424	35.87 ± 10.52
Previous CABG (%)	16	13,914	1335	9.33 ± 5.84
Prior PCI (%)	15	14,819	3770	25.44 ± 6.12
Previous valve surgery (%)	4	7097	729	10.27 ± 2.30
CVA (%)	19	15,231	1636	10.74 ± 6.85
PAD (%)	15	7459	1306	17.51 ± 5.46
COPD (%)	19	15,231	2617	17.18 ± 8.70
DM (%)	19	15,231	4133	27.14 ± 5.62
Hypertension (%)	18	15,181	11,523	75.90 ± 12.52
Dyslipidemia (%)	11	12,269	6368	51.90 ± 11.36
CKD (%)	16	14,725	2544	17.28 ± 22.89
Total Body Surface Area	4	7855	N/A	1.42 ± 0.03

Abbreviations: CABG, Coronary artery bypass graft; CAD, Coronary Artery Disease; CKD, chronic kidney disease; COPD, Chronic obstructive pulmonary disease; DM, Diabetes Mellitus; EuroSCORE, European System for Cardiac Operative Risk Evaluation; NYHA, New York Heart Association; PAD, Peripheral artery disease; STS score, Society of Thoracic Surgeon scores.

Different types of transcatheter heart valves were used as cited by the twenty studies (Table 2b, Ref. [8, 16, 17, 18, 19, 20, 21, 22, 23, 24, 25, 26, 27, 28, 29, 30, 31, 32, 33, 34]). Most of these studies used SAPIEN and CoreValve which are largely imported from western countries, while a minority used heart valves produced in Asia like the J-Valve and LOTUS. The most commonly used valve sizes among Asians were the 26-mm (38.5%) and 23-mm variants (37.9%). Most of these valves (81.8%) were installed via the transfemoral route. Prosthesis-patient mismatch occurred in 826 out of 6108 (13.5%) patients.

**Table 2b.** **Valve type and procedural characteristics**.

Study/Year	N	Valve type	Valve sizes (Most prevalent)				Access				PPM
		Brand	23 mm	26 mm	29 mm	Other	TF	TAo	TAp	Others	
Lee (2017) [16]	56	(NS)	4	25	22	5	54	1	0	1	9
Meguro (2021) [17]	5870	SAPIEN XT	2388	2260	975	247	4694	0	0	1176	Severe
		SAPIEN 3									124
		CoreValve									Moderate
		Evolut R									691
Yoon (2016) [18]	848	SAPIEN	549	299	0	0	731	0	0	117	
		CoreValve									(NS)
Miura (2017) [32]	112	SAPIEN XT	77	32	3	0	69	0	34	9	3
Liu (2018) [19]	43	J-Valve	(NS)	(NS)	(NS)	(NS)	(NS)	(NS)	(NS)	(NS)	(NS)
Tay (2021) [8]	1125	SAPIEN	343	379	222	181	910	11	72	132	
		SAPIEN 3									
		SAPIEN XT									
		CoreValve									
		Evolut R									(NS)
Chandra (2021) [33]	40	Hydra	0	18	0	22	40	0	0	0	(NS)
Takagi (2020) [34]	1613	SAPIEN	(NS)	(NS)	(NS)	(NS)	1283	0	0	330	
		CoreValve									(NS)
Saito (2021) [20]	50	LOTUS	24	0	0	26	40	10	0	0	(NS)
Yu (2018) [21]	576	SAPIEN	159	229	155	33	586	10	0	0	
		CoreValve									
		LOTUS									(NS)
Yamashita (2019) [22]	11	SAPIEN	8	2	0	1	9	2	0	0	
		CoreValve									1
Sawa (2017) [23]	64	SAPIEN XT	(NS)	(NS)	(NS)	(NS)	37	0	27	0	(NS)
Takimoto (2016) [25]	302	SAPIEN XT	193	96	10	3	200	0	99	3	(NS)
Chew (2017) [26]	59	SAPIEN	21	30	6	2	40	1	18	0	
		CoreValve									
		Evolut R									8
Liang (2021) [27]	175	SAPIEN XT	(NS)	(NS)	(NS)	(NS)	134	13	26	2	
		Venus A									
		TaurusOne									
		VitaFlow									
		J-Valve									(NS)
Sawa (2014) [24]	55	CoreValve	0	29	14	12	43	6	0	6	(NS)
Maeda (2015) [28]	15	ACURATE	(NS)	(NS)	(NS)	(NS)	10	0	0	5	
		Neo/TF									(NS)
Barbash (2015) [29]	1327	SAPIEN	200	637	413	77	1160	101	0	66	
		CoreValve									(NS)
Li (2021) [30]	1202	(NS)	(NS)	(NS)	(NS)	(NS)	1193	0	5	4	(NS)
Handa (2018) [31]	1752	SAPIEN XT	(NS)	(NS)	(NS)	(NS)	1237	0	449	66	(NS)

Abbreviations: NS, Not Specified; PPM, prosthesis-patient mismatch; TF, transfemoral; TAo, transaortic; TAp, transapical.

### 3.3 Baseline Echocardiographic Characteristics

Seventeen of the twenty studies reported their mean baseline transaortic gradient and aortic valve area as shown in **Supplementary Table 2** [8, 16, 17, 18, 20, 21, 22, 23, 24, 25, 26, 28, 29, 30, 32, 33, 34]. The mean baseline transaortic gradient was 50.93 ± 3.70 mmHg (reference range: <5 mmHg). The mean aortic valve area was 0.64 ± 0.07 ${\mathrm{cm}}^{2}$ (reference range: 2.5 to 4.5 ${\mathrm{cm}}^{2}$) [8, 16, 17, 18, 20, 21, 22, 23, 24, 25, 26, 28, 29, 30, 32, 33, 34]. Aortic regurgitation was reported in 1028 patients in thirteen studies with a weighted mean of 14.46 ± 12.56% [16, 19, 20, 22, 23, 24, 25, 28, 29, 31, 32, 33, 34]. The presence of a bicuspid valve was reported in seven studies involving 860 out of 9855 patients with a weighted mean of 8.72 ± 12.87% [8, 17, 18, 21, 26, 27, 30]. Left ventricular ejection fraction was reported in sixteen studies, and the weighted mean left ventricular ejection fraction was 60.46 ± 4.36% [8, 16, 17, 18, 19, 20, 21, 22, 25, 26, 28, 30, 31, 32, 33, 34]. Pulmonary hypertension was reported in only five studies involving 554 out of 2136 patients, and the mean prevalence of pulmonary hypertension was 25.80 ± 12.50% [8, 16, 18, 24, 33].

## 4. Outcomes

In summary, the following are the outcomes that our study have looked into in Asian studies involving TAVR. These are reflected in Table 3. Further details are given below.

**Table 3.** **Overview of post-procedural outcomes**.

Outcomes	No. of studies	Overall No. of patients	No. of events	Weighted mean
Success	15	10,989	10,470	95.28% ± 1.5%
In-hospital mortality (%)	5	3174	76	2.39 ± 0.83
30-day mortality (%)	18	13,509	224	1.66 ± 1.2
1-year mortality (%)	14	7515	655	8.79 ± 2.3
30-day stroke	15	12,704	224	1.75 ± 0.95
1-year stroke	7	2019	63	3.22 ± 1.97

### 4.1 Procedural Success

**Fig. 3.**
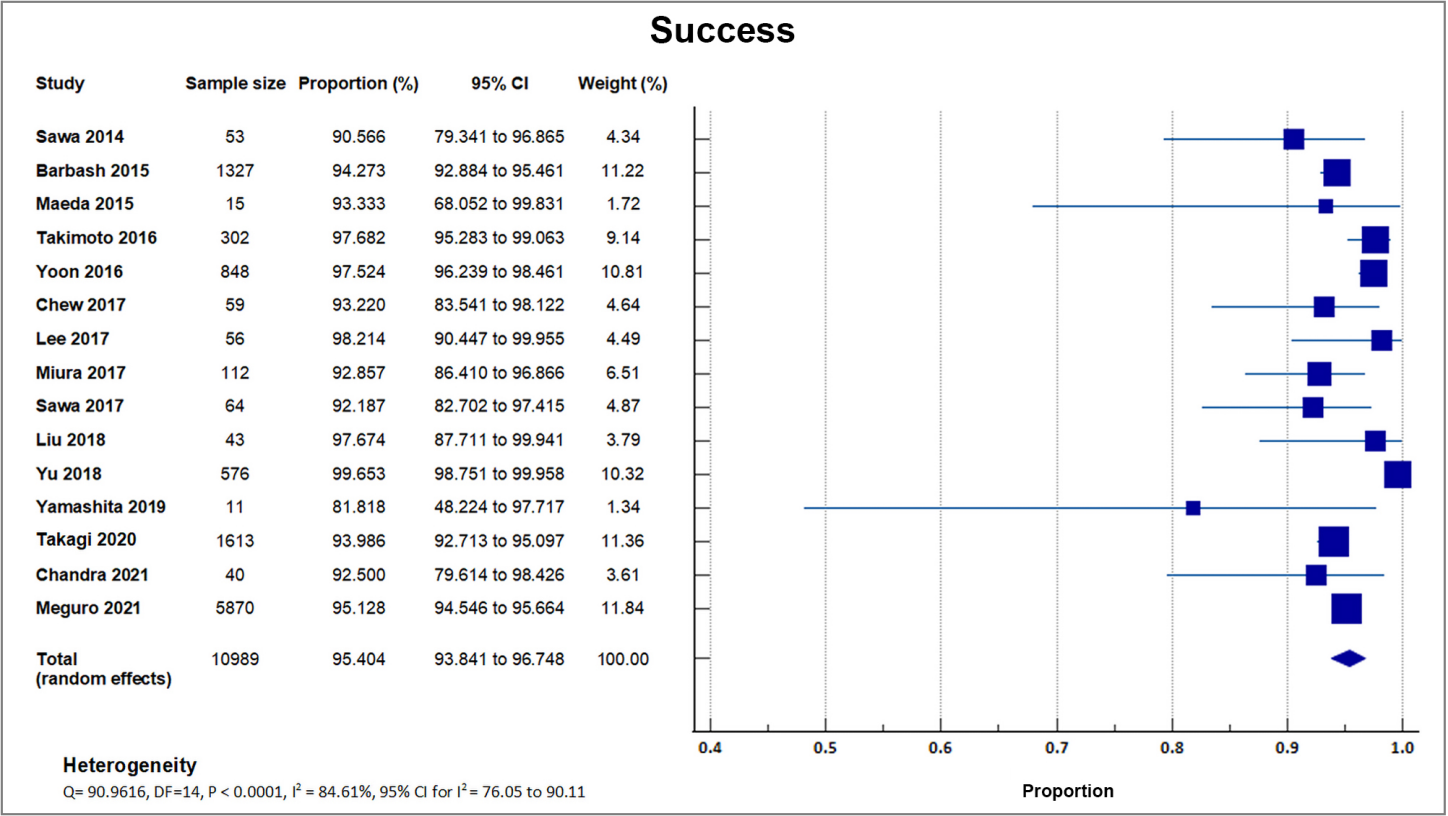
**Procedural success**. Forest plot on the rates of procedural success as an outcome of TAVR performed in Asian patients.

Fifteen registries and studies reported procedural success [16, 17, 18, 19, 21, 22, 23, 24, 25, 26, 28, 29, 32, 33, 34]. The mean success rate was 95.40% with a weighted standard deviation (SD) of 1.5% [16, 17, 18, 19, 21, 22, 23, 24, 25, 26, 28, 29, 32, 33, 34]. The highest success rate was reported in a study done by Yu in 2018 [21], with a procedural success rate of 99.7% (574/576), followed by Lee in 2017 [16], with a procedural success rate of 98.2% (55/56). A forest plot presenting all the reporting studies is shown in Fig. 3.

### 4.2 In-Hospital Mortality

**Fig. 4.**
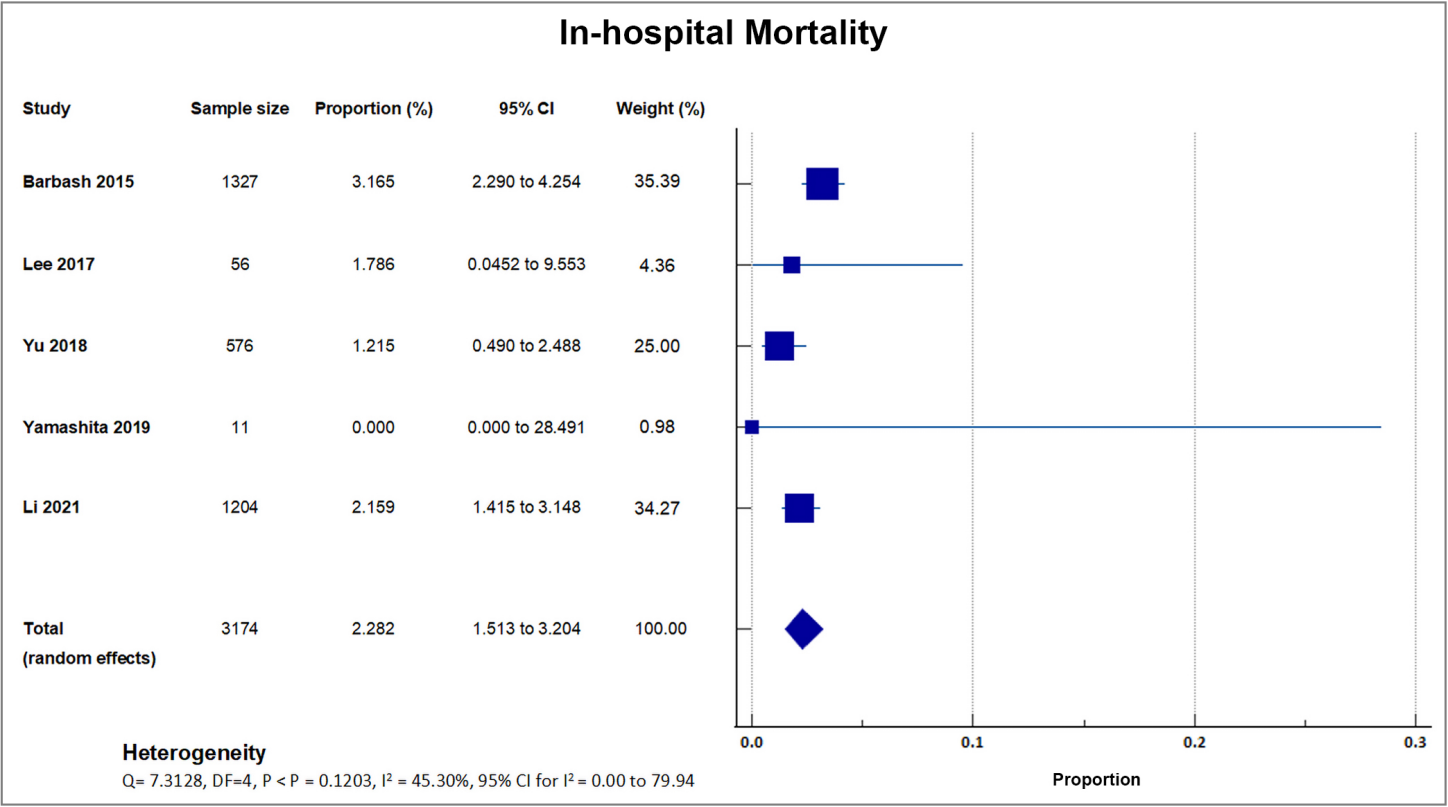
**In-hospital mortality**. Forest plot showing rates of in-hospital mortality as an outcome of TAVR performed in Asian patients.

Only five studies reported procedural and in-hospital mortality, with a total sample size of 3174 [16, 21, 22, 29, 30]. The mean in-hospital mortality rate was 2.28% [16, 21, 22, 29, 30]. Among the five studies, Barbash *et al*. [29] (2015) reported the highest in-hospital mortality (3.17%, 42/1327) in Israel. A forest plot presenting all the reporting studies is shown in Fig. 4.

### 4.3 30-Day Mortality

**Fig. 5.**
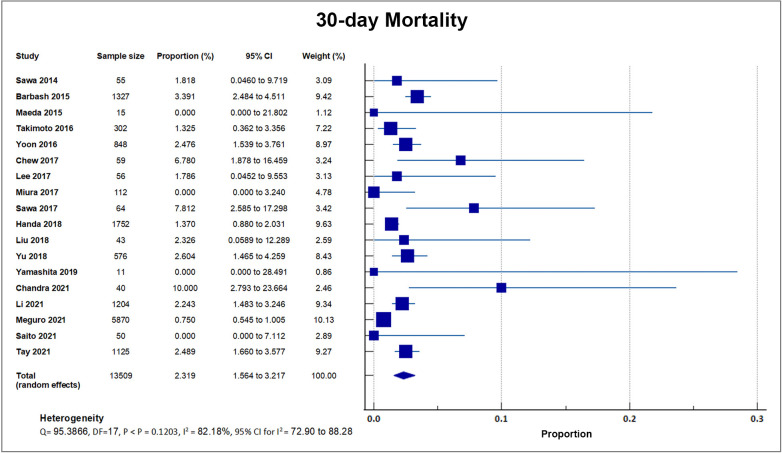
**30-day mortality**. Forest plot showing rates of 30-day Mortality as an outcome of TAVR performed in Asian patients. Abbreviations: TAVR, Transcatheter aortic valve replacement.

Eighteen studies reported that a total of 224 out of 13,509 patients died by 30 days after TAVI, giving a weighted mean for 30-day mortality rate of 1.66%, with a weighted standard deviation of 1.2% [8, 17, 18, 19, 20, 21, 22, 23, 24, 25, 26, 28, 29, 30, 31, 32, 33]. Four small center trials and registries with sample sizes ranging from only 15–50 recorded a 0% mortality. These four studies had a weight ranging from 0.08% to 0.37%. A forest plot presenting all the reporting studies is shown in Fig. 5 [20, 22, 28, 32].

### 4.4 One-Year All-Cause Mortality

**Fig. 6.**
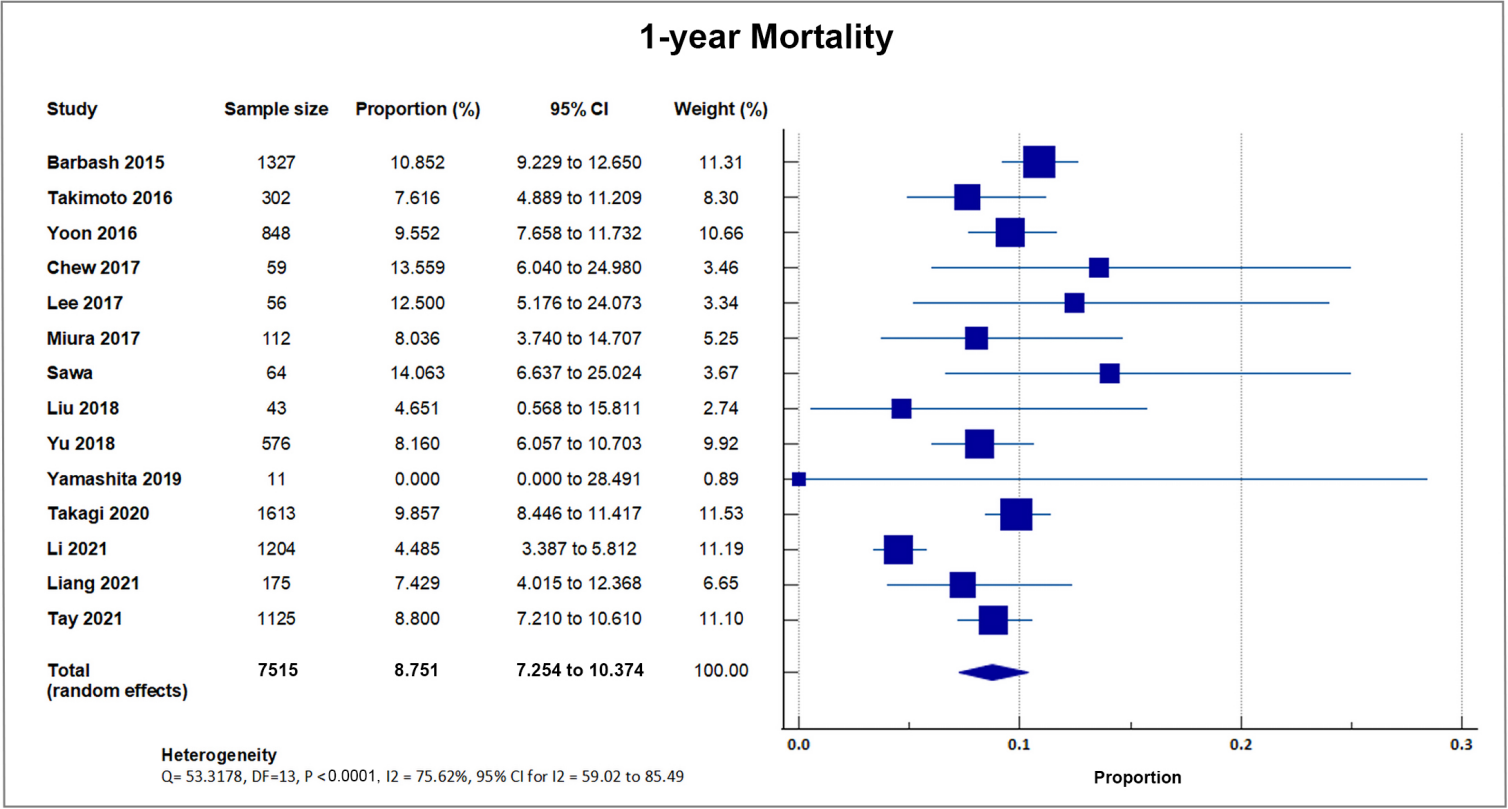
**1-year mortality**. Forest plot showing rates of one-year mortality as an outcome of TAVR performed in Asian patients. Abbreviations: TAVR, Transcatheter aortic valve replacement.

Fourteen studies reported one-year all-cause mortality with a total of 655 out of 7515 patients bringing the weighted mean at 8.79, SD 2.3% [8, 16, 18, 19, 21, 22, 24, 25, 26, 27, 29, 30, 32, 34]. A forest plot presenting all the reporting studies is shown in Fig. 6.

### 4.5 Stroke Incidence

**Fig. 7.**
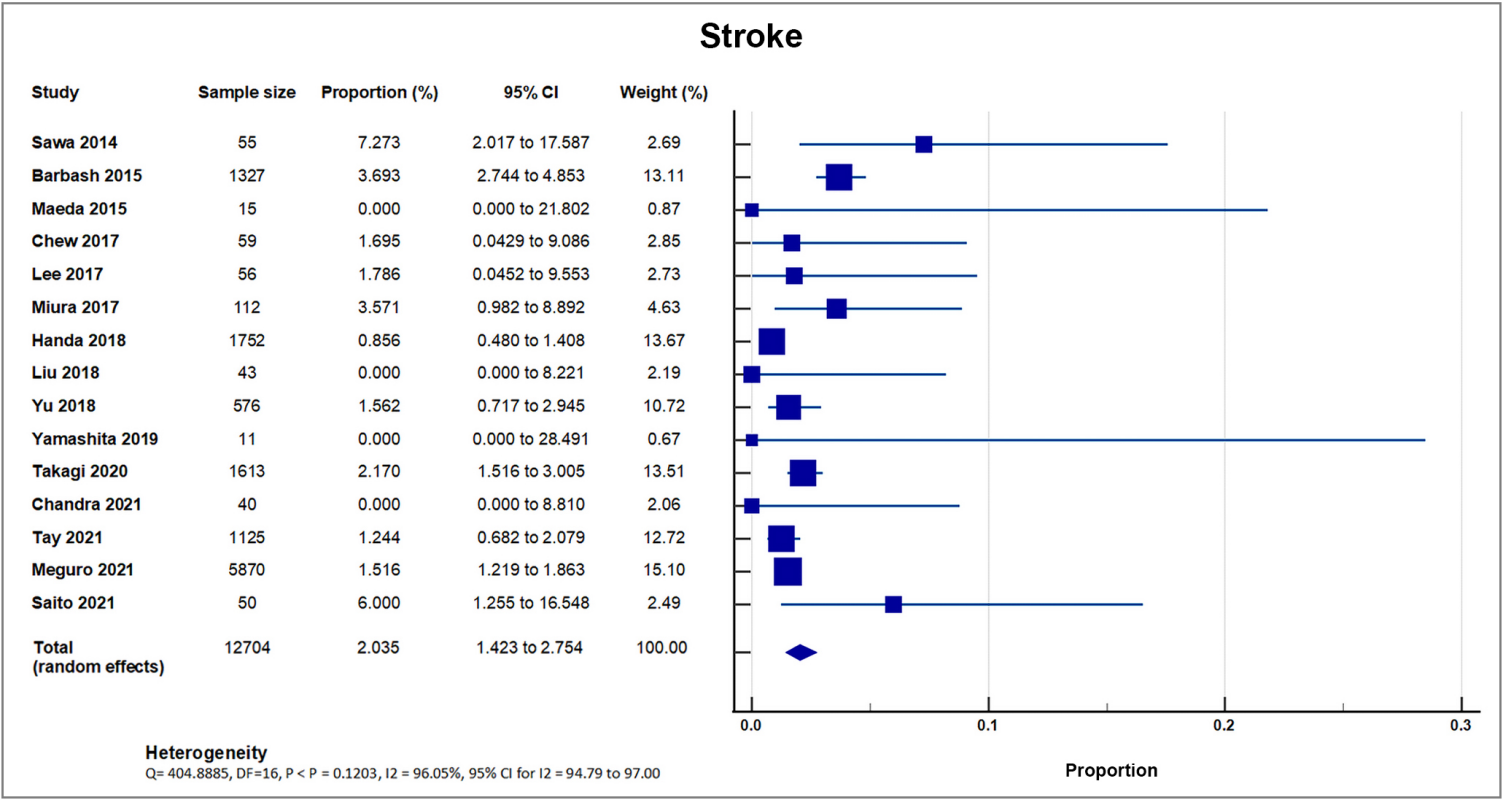
**Stroke**. Forest plot showing rates of stroke as an outcome of TAVR performed in Asian patients. Abbreviations: TAVR, Transcatheter aortic valve replacement.

All included studies reported stroke events as outcomes. Out of 20, three small sample studies and registries with sample sizes ranging from only 15–50 reported no occurrence of stroke [19, 28, 33]. 17 studies reported occurrence of stroke, with a total of 303 out of 15,297 patients, with a weighted mean of 1.98%, SD 1.49% [8, 16, 17, 18, 20, 21, 22, 23, 24, 25, 26, 27, 29, 30, 31, 32, 34]. 15 studies reported the incidence of stroke within 30 days as an outcome, with a mean of 1.75% [8, 16, 17, 19, 20, 21, 22, 24, 26, 29, 30, 31, 32, 33, 34]; and seven studies reported the incidence of stroke in one year as an outcome, with a mean of 3.22%. A forest plot presenting these 15 studies is shown in Fig. 7 [8, 19, 21, 22, 23, 27, 30].

### 4.6 Complications

Table 4 (Ref. [8, 16, 17, 18, 19, 20, 21, 22, 23, 24, 25, 26, 27, 28, 29, 30, 31, 32, 33, 34]) shows an overview of the acute procedural complications reported by the included studies as outcomes.

**Table 4.** **Acute procedural complications**.

Complication	No. of studies	Overall no. of patients	No. of events	Weighted mean
AKI (%)	[8, 16, 18, 19, 20, 23, 26, 28, 29, 31, 32, 33, 34]	7104	489	6.88 ± 5.71
Major vascular complications (%)	[8, 16, 18, 19, 20, 21, 22, 24, 25, 26, 27, 30, 31, 32, 33]	6408	167	2.58 ± 2.54
Major bleeding (%)	[8, 16, 18, 19, 20, 21, 22, 24, 25, 26, 28, 29, 30, 31, 32, 33, 34]	9188	359	3.88 ± 3.74
Perivalvular Aortic Regurgitation, Moderate to Severe (%)	[8, 16, 17, 18, 19, 20, 22, 24, 25, 26, 28, 29, 33, 34]	11,410	1720	15.07 ± 9.58
Permanent Pacemaker Insertion (%)	[8, 16, 17, 18, 19, 20, 21, 23, 24, 26, 27, 28, 29, 30, 31, 33, 34]	15,110	1177	7.76 ± 4.6
New Onset Atrial Fibrillation (%)	[20, 26, 27, 30, 34]	3099	75	2.42 ± 0.03

Abbreviations: AKI, Acute kidney injury.

#### 4.6.1 Acute Kidney Injury 

**Fig. 8.**
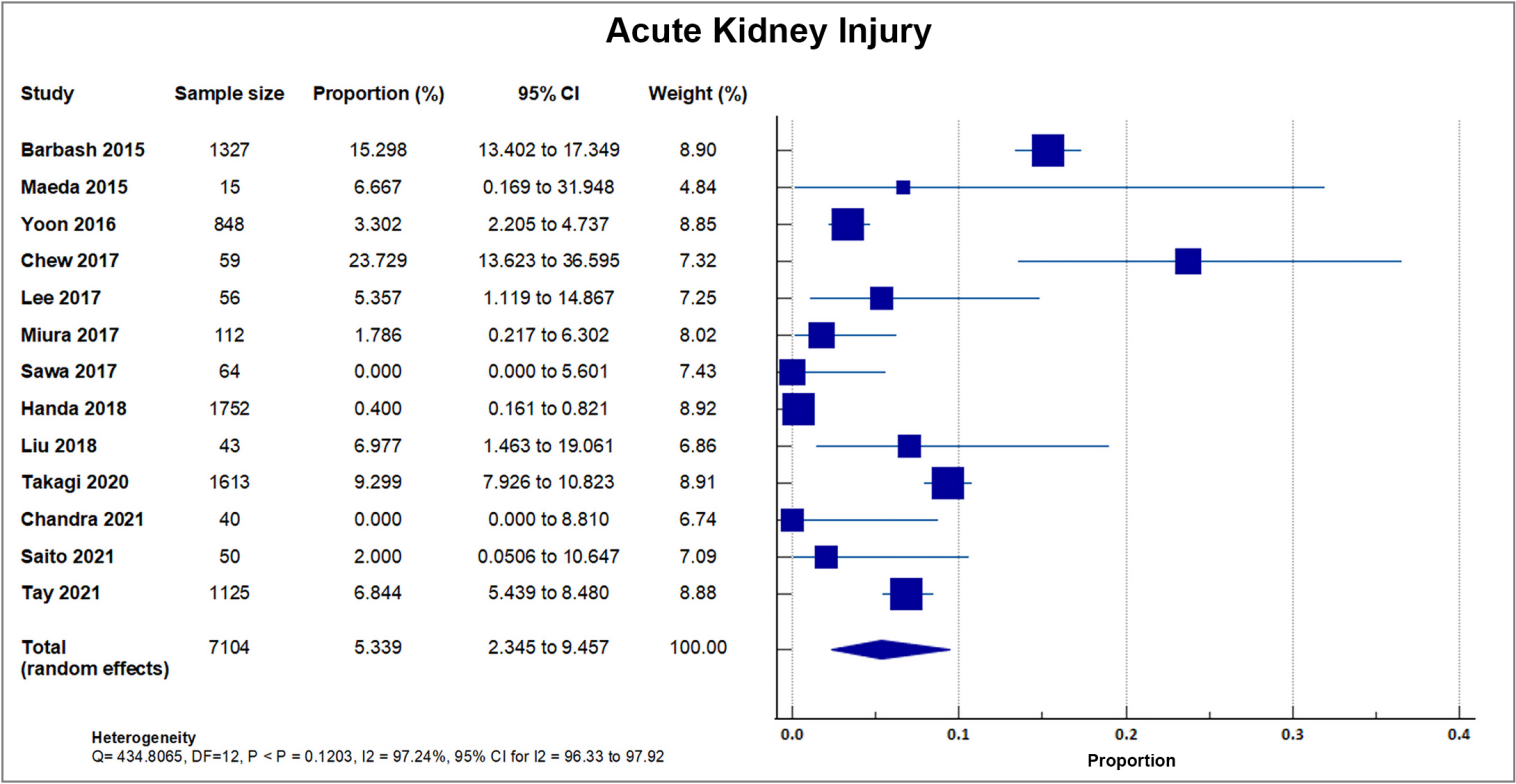
**Acute kidney injury (AKI)**. Forest plot showing rates of AKI as an outcome of transcatheter aortic valve replacement (TAVR) performed in Asian patients.

Thirteen studies reported Acute Kidney Injury (AKI) in 489 out of 7104 patients, with a mean AKI rate of 6.88, SD 5.71% [8, 16, 18, 19, 20, 23, 26, 28, 29, 31, 32, 33, 34]. The highest percentage was reported in a single center study in Singapore with 23.7% (14/59). Two studies reported 0% incidence of AKI [23, 33]. A forest plot presenting all the reporting studies is shown in Fig. 8.

#### 4.6.2 Major Vascular Complications

**Fig. 9.**
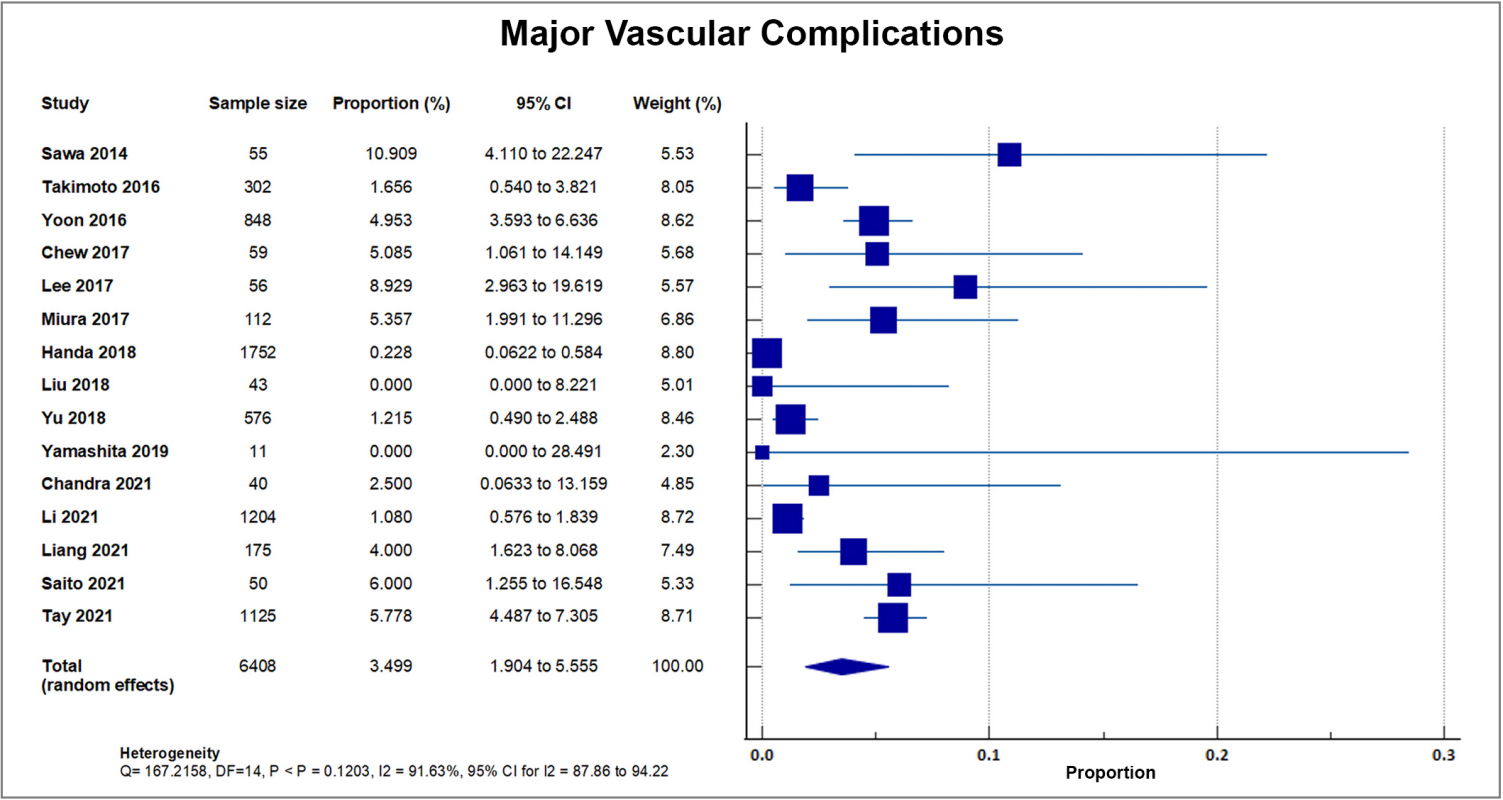
**Major vascular complication**. Forest plot showing rates of major vascular complications as an outcome of TAVR performed in Asian patients. Abbreviations: TAVR, Transcatheter aortic valve replacement.

As reported by 15 out of 20 studies, a total of 167 out of 6408 patients suffered from major vascular complications, accounting for an overall rate of 2.48%, SD 2.54%. A forest plot presenting all the reporting studies is shown in Fig. 9 [8, 16, 18, 19, 20, 21, 22, 24, 25, 26, 27, 30, 31, 32, 33].

#### 4.6.3 Major Bleeding

**Fig. 10.**
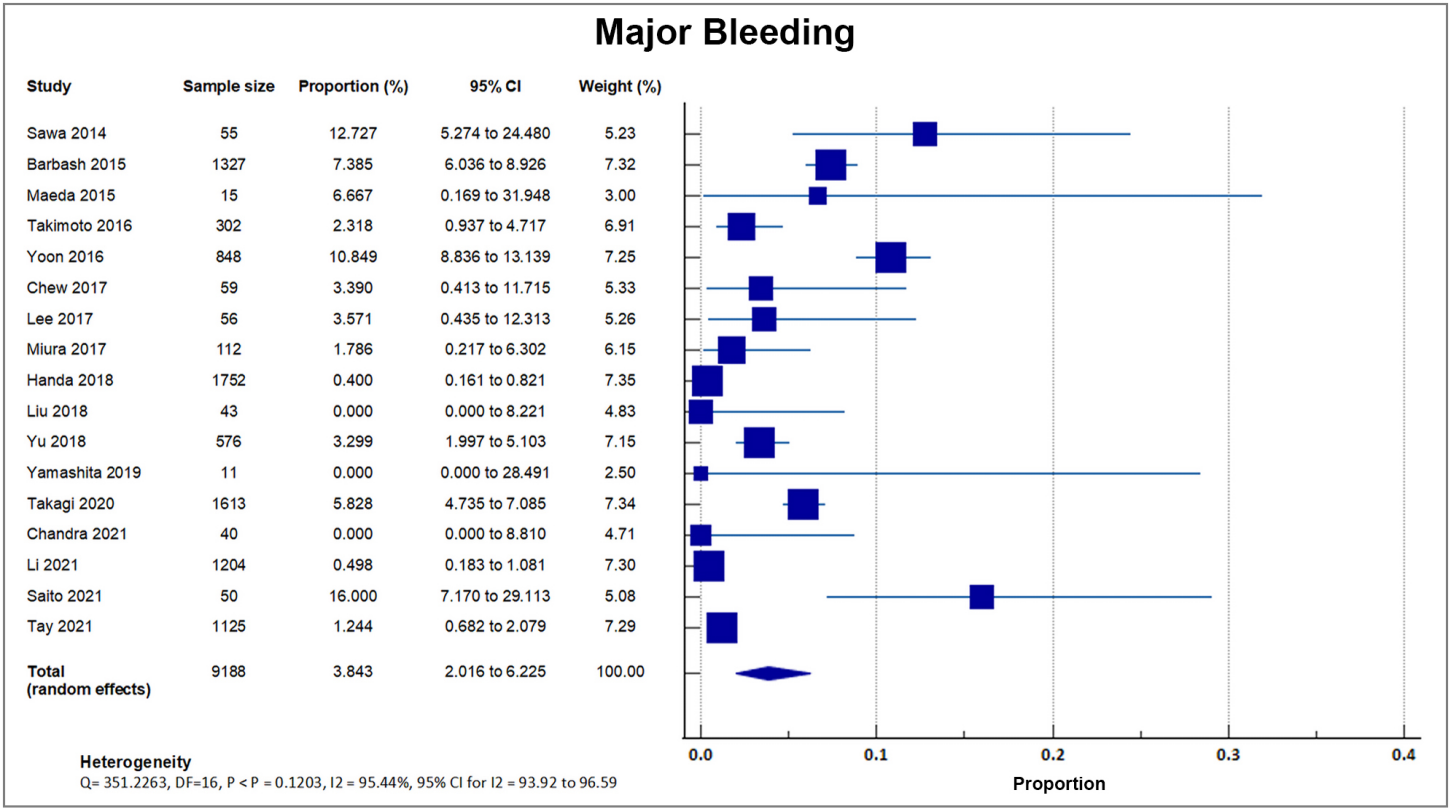
**Major bleeding**. Forest plot showing rates of major bleeding as an outcome of TAVR performed in Asian patients. Abbreviations: TAVR, Transcatheter aortic valve replacement.

Seventeen studies reported that during the first 30 days after TAVI, 359 out of the total of 9188 patients suffered from major bleeding as defined in VARC-2, accounting for an overall bleeding rate of 3.88 ± 3.74%. A forest plot presenting all the reporting studies is shown in Fig. 10 [8, 16, 18, 19, 20, 21, 22, 24, 25, 26, 28, 29, 30, 31, 32, 33, 34].

#### 4.6.4 Perivalvular Aortic Regurgitation

**Fig. 11.**
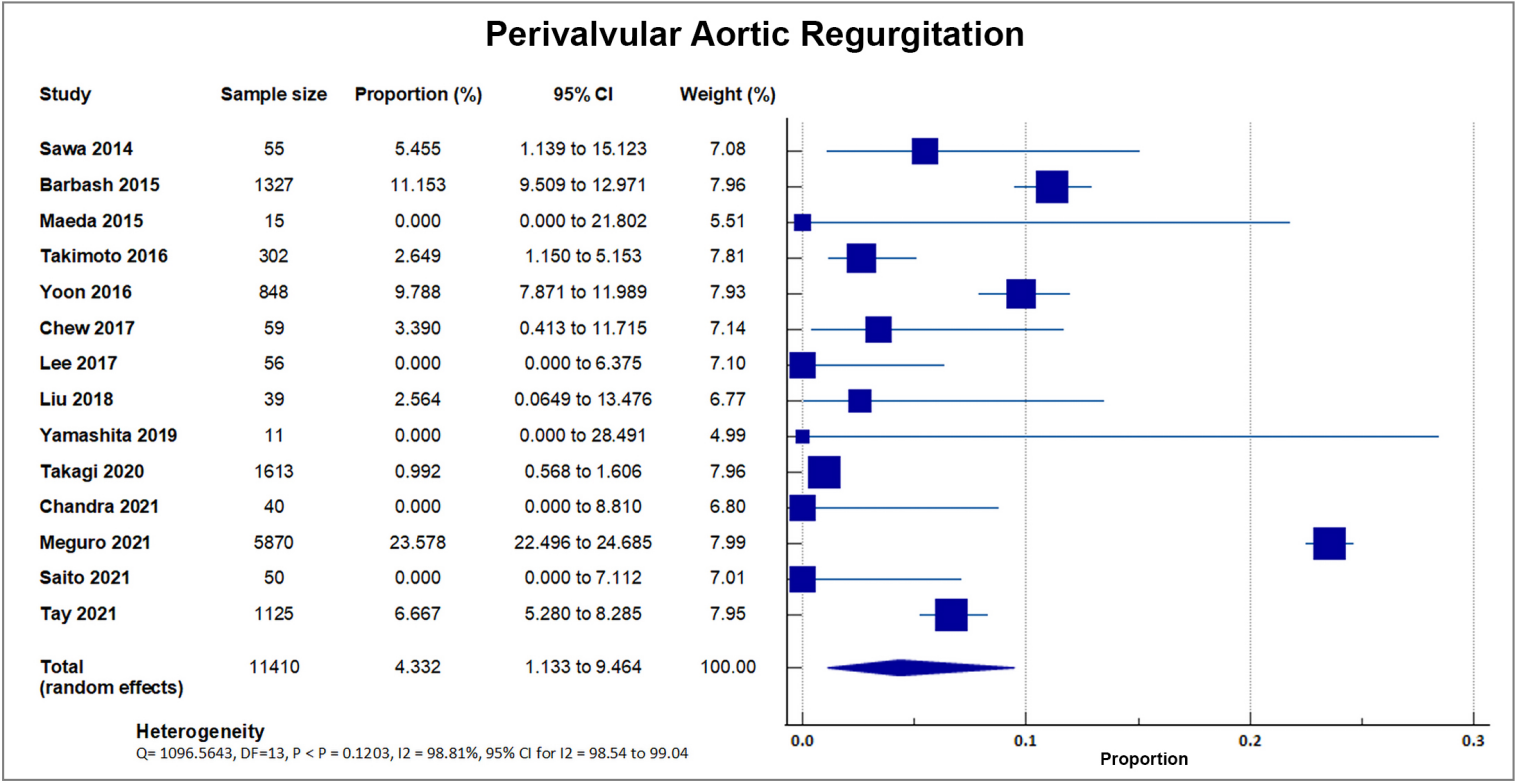
**Perivalvular aortic regurgitation**. Forest plot showing rates of perivalvular aortic regurgitation as an outcome of transcatheter aortic valve replacement (TAVR) performed in Asian patients.

Fourteen out of 20 studies reported moderate to severe perivalvular aortic regurgitation. A total of 1720 out of 11,410 patients experienced postprocedural aortic regurgitation, accounting for a weighted rate of 15.07, SD 9.58% [8, 16, 17, 18, 19, 20, 22, 24, 25, 26, 28, 29, 33, 34]. J-TVT, a large registry developed by 4 Japanese academic societies with 5870 enrolled patients, reported the highest percentage of paravalvular leakage at 23.58% with a weight of 51.43%. A forest plot presenting all the reporting studies is shown in Fig. 11.

#### 4.6.5 Permanent Pacemaker Implantation

**Fig. 12.**
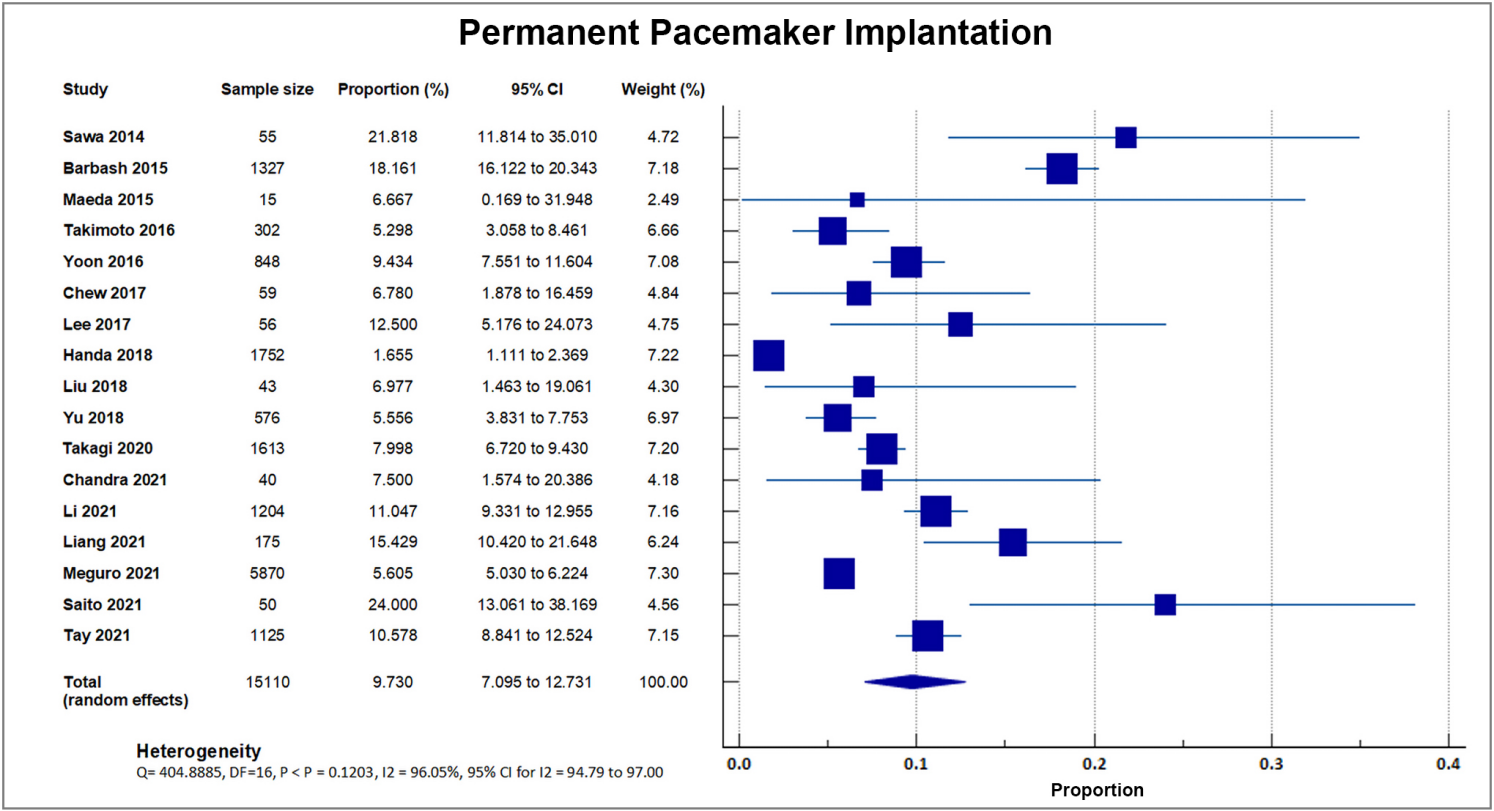
**Permanent pacemaker insertion**. Forest plot showing rates of PPI as an outcome of TAVR performed in Asian patients. Abbreviations: PPI, permanent pacemaker implantation; TAVR, Transcatheter aortic valve replacement.

Seventeen studies reported a post-procedural need for permanent pacemaker implantation. A total of 1177 out of 15,110 patients required permanent pacemaker implantation, accounting for an overall rate of 7.76%, SD 4.6% [8, 16, 17, 18, 19, 20, 21, 23, 24, 26, 27, 28, 29, 30, 31, 33, 34]. A forest plot presenting all the reporting studies is shown in Fig. 12.

#### 4.6.6 New-Onset Atrial Fibrillation

Five studies reported on the incidence of new onset atrial fibrillation (Table 4). Of the 3099 patients, 75 (2.42%) experienced new-onset, postoperative atrial fibrillation [20, 26, 27, 30, 34].

## 5. Discussion

### 5.1 Growth and Challenges of TAVR in Asia

**Fig. 13.**
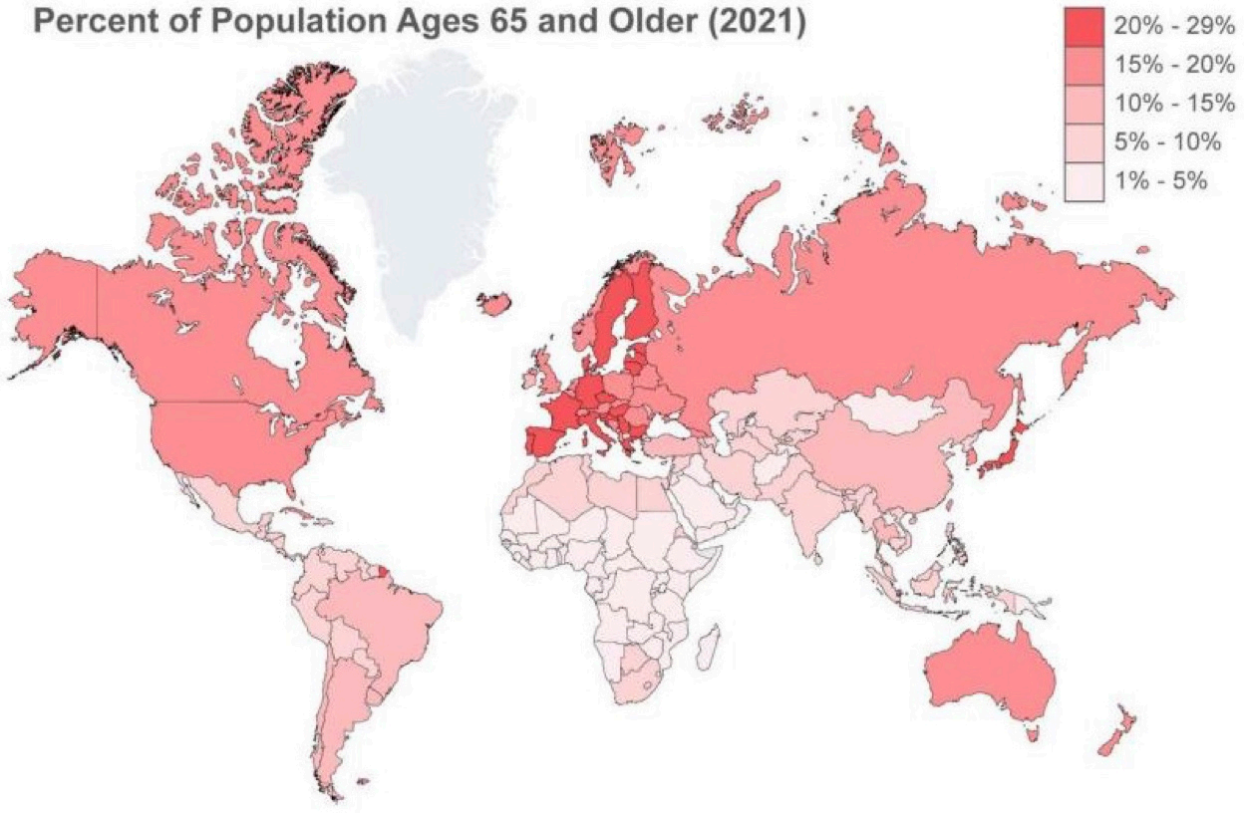
**Percentage of the population ages 65 and older**.

It was only after the publication of randomized trials demonstrating TAVI as a solid and unquestionable treatment modality for aortic stenosis that case numbers begin to rise in Asia [7]. Demographics play a role in the rapid expansion of TAVI centers in Asia [35]. The elderly population, usually those aged >65 years old, are among the most affected population who receive TAVI procedure more often. This trend is observed in Japan, with the highest incidence of AS due to its elderly population, followed by Hong Kong, South Korea, Taiwan, Singapore, and China [7] (see Fig. 13). In addition, this procedure has gained commercial approval in Japan in 2013, with patients given reimbursements for TAVI-related costs [7]. However, this is not the case for most other Asian Pacific countries, wherein higher out-of-pocket fees due to a lack of medical reimbursements can potentially limit its accessibility [3]. The estimated procedural cost for TAVI is approximately USD 35,000 in India and can be as high as USD 47,000 in Thailand, while SAVR in Thailand costs almost USD 17,000 [4]. Although studies have shown that TAVI causes increased quality-adjusted survival by 15 to 27 years and lower long-term costs than SAVR, the lack of medical reimbursement for TAVI can unfortunately mask its cost-effectiveness over SAVR on high-risk patients [36]. Therefore, TAVI procedures can appear to be very expensive, with some Asian patients opting for SAVR instead as a cheaper option [7]. Aside from the cost factor, lack of specialty centers and lack of government policies have slowed the growth of TAVI in India and other low to middle-income countries in Asia [4]. With those factors said, the practice pattern and outcome of medical devices following their regulatory approval may differ by country [37]. In 2022, Kaneko *et al*. [38] compared post-approval national clinical registry data on TAVI between the United States (US) and Japan on patient characteristics, periprocedural outcomes, and the variability of outcomes as a part of a partnership program (Harmonization-by-Doing) between the two countries. Both countries obtained excellent outcomes, although the Japanese had lower 30-day mortality and major morbidity. Since its slow start a decade ago, TAVI has come a long way in Asia [7]. TAVI is now performed in almost 300 centers throughout Northeast Asia, Southeast Asia, and the Indian subcontinent [7].

### 5.2 Outcomes in Asia versus Europe and the US

Compared to their American and European counterparts, Asian patients who underwent TAVI experienced less 1-year all-cause mortality, bleeding, and vascular complications; but had more postprocedural aortic regurgitation. 30-day mortality and incidence of stroke, AKI, and the need for a permanent pacemaker were similar. Detailed comparisons of Asian figures for the key outcomes compared to those found in America and Europe are found below.

### 5.3 30-Day Mortality

Our analysis of eighteen registries that reported the 30-day mortality revealed a weighted average of 1.66 ± 1.21%. This finding was similar to the 2021 data from the US STS-American College of Cardiology Transcatheter Valve Therapy Registry with a 30-day all-cause mortality of 2.5% [39] but substantially lower when compared to the United Kingdom data at 5.14% [40]. These findings may be due to similar age, comorbidity status and STS risk scores between the US, UK and Asian cohorts [39, 40, 41]. The smaller sample size of patients enrolled in Asian studies compared the US or UK registries is a limitation to making comparing between these three populations.

### 5.4 1-Year Mortality

Our analysis of the fourteen reports which included 1-year mortality following TAVR yielded a weighted mean of 8.79 ± 2.3%, which is significantly lower than the data reported by western registries. Pooled Rotterdam-Milan-Toulouse in Collaboration (PRAGMATIC) 2015 [Milan, Rotterdam, Toulouse] reported a 1-year all-cause death rate of 18.5% [42], Swiss-TAVI 2019 reported a rate of 13.0% [43], and the US STS-American College of Cardiology Transcatheter Valve Therapy Registry reported a rate of 12.6% [44].

There have been no large-scale studies directly comparing outcome differences in TAVI recipients between Asian and Western populations; however, a recent report on racial disparities in outcomes from the TVT registry showed that the adjusted 1-year mortality rate was significantly lower among patients of Asian/Native American/Pacific Islander descent than when compared to White patients [45]. However, of the 70,221 patients included in the report, Asian patients only comprised <2%, making direct comparisons difficult. The data retrieved for 1-year mortality appears to be more heterogenous than that for the 30-day mortality, which suggests that more factors, including non-cardiac deaths may have confounded the contribution of TAVR to this statistic. Similar to the 30-day mortality statistic, the difference in Asian data should be taken in careful consideration with the smaller sample size and potential underreporting in Asian registries.

### 5.5 Stroke

Ischemic stroke is a feared complication associated with TAVI. TAVI is associated with a significantly higher ischemic cerebrovascular events [CVE] risk in the early phase (hazard ratio (HR) 5.35 [95% CI 3.50–8.17]; *p *< 0.001) but not in the late phase (HR 1.17 [95% CI 0.94–1.46]; *p* = 0.15) [46]. In the meta-analysis by Eggebrecht *et al*. [47], fifty-three studies were analyzed, including 10,037 patients undergoing transfemoral, transapical or trans-subclavian TAVI for native aortic valve stenosis. The overall 30-day stroke/ TIA was 3.3 ± 1.8%, with the majority being major strokes (2.9 ± 1.8%). Taking into consideration the standard deviation, these findings are consistent with the result of our meta-analysis. The etiology of stroke after TAVI is multifactorial and includes embolism of valvular material during balloon valvuloplasty, device manipulation across an atheromatous aorta, and atrial fibrillation. In Optimized transCathEter vAlvular iNtervention (OCEAN-TAVI), independent predictors of 1- to 30-day Cerebrovascular events (CVE) were paroxysmal atrial fibrillation and index aortic valve area (iAVA) after TAVR [34]. Consequently, independent predictors of 30-day cerebrovascular events (CVE) were prior stroke, paroxysmal atrial fibrillation (PAF), and coronary artery disease [34]. iAVA independently predicted 24-hour CVEs using multivariate analysis, at receiver operator curve derived cut-off value of 0.40 ${\mathrm{cm}}^{2}$/$m^{2}$.

### 5.6 Acute Kidney Injury 

In the thirteen publications that reported AKI with 7104 patients, the event occurred in 489 patients within the first 30 days from the procedure resulting in a weighted mean of 6.88 ± 5.71%. This rate is relatively lower compared to US cohorts. AKI is a frequent complication after TAVI and affects outcome and survival [48]. Previous studies showed that patients have an increased risk of postoperative AKI after TAVI, but whether differences in patient risk profiles confounded the results is unclear [49]. In the recent work by Julien *et al*. [50], out of 107,814 patients who had TAVI in the US, 11,566 (10.7%) experienced postprocedural AKI. Among patients who developed AKI, 10,220 (9.5%) developed stage 1 AKI, 134 (0.1%) stage 2 AKI, and 1212 (1.1%) stage 3 AKI. A similar study by Abbas *et al*. [51], utilizing the US National In-patient registry, reported a similar percentage at 11.5% (20,045/173,760). The 30-day mortality rate for AKI patients after TAVI is 7.8–29%. This rate is two to eight times higher than those without AKI. Hospital length of stay is also increased 2.5 times in patients with AKI. The mechanism is most likely a combination of prerenal azotemia and direct nephrotoxic influences leading to renal ischemia and acute tubular necrosis [48]. Predictors of AKI include male sex, chronic kidney problem [52], heart failure, AF, transapical approach, and cardiac and vascular complications. Transfusion with packed red blood cells was found to be an independent predictor of AKI, and it predicts both the 30-day and cumulative mortality [53].

### 5.7 Bleeding and Vascular Complications

Post-TAVI bleeding, major or life-threatening, increases 30-day postoperative mortality [54]. Seventeen publications reported major bleeding, and out of 9188 patients, the event occurred in 359 with a mean of 3.88 ± 3.74%. This rate is substantially lower compared to the early data by Kochman *et al*. [55], in which serious bleeding events occurred in 19% of cohorts, of which 12.4% had major bleeding. Transapical access and preexisting AF independently correlated with TAVI-associated bleeding, likely because of AF-related anticoagulation [54]. Furthermore, the study of Kochman *et al*. [55] revealed that trans-subclavian access and diabetes are independent predictors of significant bleeding events.

Patients undergoing TAVI between 2011 to 2016 showed a vascular complication rate of 9.3% (n = 3257) and an in-hospital bleeding event rate of 7.6% (n = 2651). Rates of vascular complications and bleeding events decreased over time (*p* for trend test <0.0001) [56]. Randomized clinical trials and clinical evidence on post-TAVI bleeding in Asian patients are still scarce. This is an important gap in knowledge as East Asian patients are known to have increased bleeding risk during antithrombotic therapy when compared with White patients (also known as the “East Asian paradox”) [56].

Vascular complications are one of the major concerns during TAVR, primarily due to using large bore sheaths to establish adequate access [56]. In the PARTNER (Placement of AoRTic TraNscathetER Valve) Trial, sixty-four patients (15.3%) had major vascular complications, and 50 patients (11.9%) had minor vascular complications within 30 days of the procedure [1]. Most TAVI procedures performed in Asia used the transfemoral approach [8]. Transfemoral access use was similar in the US (US-TVT) and Japan (Japan-TVT) at rates of 90.9% and 88.7%, respectively [38, 57]. However, transapical access was more commonly done in Japan than in the US (20.1% versus 42.5%; *p *< 0.001) [38]. In our analysis involving 6408 patients from sixteen publications, 169 patients had major vascular complications with a weighted mean of 2.58 ± 2.54%, substantially lower than the previously mentioned studies. One study had significantly higher rates of complication than the other study and reports that over 280 patients out of 1327 (21%) had post-procedural vascular complications [34]. In the study of Czerwińska-Jelonkiewicz *et al*. [58], vascular complications, which occurred in 30 days after TAVI, predicted late mortality (*p* = 0.036). They concluded that TAVI patients with anemia and diabetes mellitus are at high risk for vascular complications [58].

### 5.8 Paravalvular Aortic Regurgitation

Aortic regurgitation after TAVI is linked to adverse outcomes, and the most common cause is PVL. PVL occurs in undersized valves, markedly elliptical annulus geometry, and if the prosthetic valve is not apposed properly to the native valve due to extensive calcification or malposition [59]. The study reveals comparable rates of moderate to severe paravalvular aortic regurgitation compared to the reported incidence in Western Countries, i.e., PRAGMATIC 2015 (2.3%) and Swiss-TAVI (5.0%) [42, 43]. The use of similar types of valves between Asian and Western groups may explain the similar rates of PVL, however, the differences in anatomy (i.e., incidence of bicuspid valve and smaller valve diameters) and center and surgeon experience have to be considered [60].

### 5.9 Need for Permanent Pacemaker

The mean rate of permanent pacemaker insertions post-TAVI in the included Asian registries is comparable to the reported rates of permanent pacemaker insertion in PRAGMATIC 2015 (15.6%) and Swiss-TAVI (18.5%) [42, 61].

## 6. Conclusions

Since its inception, TAVI has grown tremendously, and various registries report constantly declining mortality and complication rates with the procedure. The demand for TAVI in Asia is expected to rise due to its aging population. Our research suggests similar post-TAVI mortality and complications in Asian countries compared to the US and Europe. One-year mortality, bleeding, and vascular complications occurred less frequently, but postprocedural aortic regurgitation was more common. Anatomical differences and disparities in access to technical expertise and health resources may play a major role in these differences. More studies with a greater sample size focusing on the clinical outcomes and anatomic differences among Asians are needed to make a more robust comparison between Asian and Western populations. The significant socioeconomic barriers to TAVI access must be addressed for broader implementation of the procedure in Asia.

### Strengths and Limitations

This meta-analysis explored TAVI outcomes and complications in Asia, which features cohorts from Hong Kong, Japan, the Philippines, Singapore, Taiwan, South Korea, and Israel. To our knowledge, this paper is the largest aggregated report available at this time of writing. However, the total of 15,297 patients this study described still does not compare to the sample size reported by Western registries in the US and Europe; as such, making direct comparisons with this disparity in sample size is challenging. Direct comparison using meta-analysis with other randomized western registries is limited. Furthermore, data gathered from registries and trials concentrated on high-income Asian economies in the region and may not accurately represent the entire Asian population.

Adoption of TAVI in Asia has been slow, particularly among developing countries with a significant infrastructural gap that hinders more widespread use of the procedure [2, 41]. A closer look at these disparities is highly recommended for future research.

## Abbreviations

TAVR, Transcatheter Aortic Valve Replacement; TAVI, Transcatheter Aortic Valve Implantation; EuroSCORE, European Systems for Cardiac Operations Risk Evaluation; STS Score, Society of Thoracic Surgeons Score; NYHA, New York Heart Association; CAD, Coronary Artery Disease; CABG, Coronary artery bypass graft; PAD, Peripheral artery disease; COPD, Chronic obstructive pulmonary disease; DM, Diabetes Mellitus; CKD, chronic kidney disease.

## Supplementary Material

Supplementary material associated with this article can be found, in the online version, at https://doi.org/10.31083/j.rcm2403079.
